# Processing of Haynes^®^ 282^®^ Alloy by Direct Energy Deposition with Arc and Wire

**DOI:** 10.3390/ma16041715

**Published:** 2023-02-18

**Authors:** Manuela Zinke, Stefan Burger, Sven Jüttner

**Affiliations:** 1 Institute of Materials and Joining Technology, Otto von Guericke University, 39106 Magdeburg, Germany; 2 Bionic Production GmbH, 21339 Lueneburg, Germany

**Keywords:** additive manufacturing, Haynes^®^ 282^®^, CMT process, shielding gas, cooling time, mechanical properties, microstructure, PWHT

## Abstract

Direct energy deposition with arc and wire (DED-AW) is a versatile, low-cost, and energy-efficient technology for additive manufacturing of medium- and large-sized metallic components. In this study, the effects of arc energy and shielding gas in cold metal transfer (CMT) welding of walls and blocks on cooling time, mechanical properties, and macro- and microstructure have been studied using precipitation-hardenable Ni-based superalloy Haynes^®^ 282^®^. The arc energy and consequently the cooling rate were varied by changing the wire feed rate and the travel speed. As expected, increasing the arc energy leads to higher cooling times for the walls. Due to the 2D thermal conduction, the thin walls cool down much slower than multi-layer welded blocks, but this reduces the strength values only very slightly. While the walls have no sensitivity to the occurrence of unacceptable seam irregularities, the multi-layer blocks show isolated seam defects, such as hot cracks or lack of fusion. Despite shielding gas variation, the as-welded blocks show acceptable strength properties at room temperatures (RT) and impact values at RT and −196 °C. However, the use of an N-containing shielding gas results in lower elongation and notched bar impact energy. Precipitation-hardened specimens tested at 871 °C exhibit a similar strength level to transverse tensile specimens of gas metal arc welding (GMAW) welded joints on 12.7 mm thick plates with fracture in the weld metal.

## 1. Introduction

In additive manufacturing (AM), parts are made from 3D model data by depositing layer by layer of material [1]. It consists of seven process categories, including directed energy deposition (DED) and powder bed fusion (PBF), both of which enable the generation of near-full-density metal parts. In contrast to powder bed processes, in directed energy deposition (DED), the feedstock (powder or wire) is melted by means of an arc, laser beam, or electron beam and introduced directly into the molten pool [2]. In the literature, the DED arc and wire technology (DED-AW) is also often referred to as wire arc additive manufacturing (WAAM).

Modern high-temperature applications such as in gas turbines for the aerospace and energy sectors would not operate safely without utilizing Ni-based superalloys, which exhibit outstanding creep and oxidation resistance at elevated temperatures [3]. These superalloys are strengthened by solid-solution and precipitation hardening brought by two main types of precipitates: gamma-prime (γ′) and gamma-double-prime (γ″) [4].

HAYNES^®^ 282^®^ (HY282, Ni-19Cr-10Co-8.5Mo-1.5Al-2.1Ti) is a precipitation-hardenable nickel-based superalloy developed for the above high-energy applications in the operating temperature range of 649 to 927 °C. It achieves its properties through a relatively low γ′-phase volume fraction of 19% and by solid-solution strengthening through additions of Mo, Cr and Co. In addition, Co also influences the solubility of the elements in the gamma matrix, Cr provides a high oxidation and hot corrosion resistance, and Mo is responsible for the excellent creep resistance. The microstructure of this alloy also includes MC, M_6_C, and M_23_C_6_ carbides, which also play a role in strengthening [5,6]. The welds of HY282 also show excellent solidification and HAZ liquid cracking resistance, which has been demonstrated by Varestraint testing and Gleeble hot ductility tests [7]. Many studies [8,9,10,11,12] report on the successful processability of this material by conventional welding.

In terms of the additive manufacturing of HY282, the investigations focus on electron or laser beam powder bed fusion (PBF) [13,14,15,16,17] and DED with laser [18]. Few publications are available for WAAM with this alloy, although it is of interest for medium and large-size components in high-temperature applications of aircraft engines and industrial gas turbines. Thus, in [19,20], the influence of different build-up strategies for the gas tungsten arc welding (GTAW) of block and cylinder parts of this material on the recrystallization behavior and grain structures, as well as different heat treatment strategies on the carbide development and γ′-precipitation, were investigated. Investigations on the influence of different arc energies and shielding gas mixtures during additive CMT arc welding with HY282 on the mechanical properties and microstructure, on the other hand, are not yet available.

Cold metal transfer (CMT) is a modified GMAW process that provides low arc energy and controlled material deposition. It has a low dilution rate, less spattering, and high energy density and shows good suitability for additive manufacturing of wall structures with different Ni-based wire electrodes [21,22,23,24]. However, when producing CMT multi-track depositions with stringer beads, lack of fusion and hot cracks can occur [25]. To improve the wetting behavior of the viscous nickel melt and reduce the risk of lack of fusion, other GMAW modes of metal transfer, such as pulsed arc or mixed shielding gases with argon, helium, hydrogen, or carbon dioxide, can be used. He and H_2_ ensure a higher arc temperature, which means that larger weld penetrations and higher travel speeds can be achieved. CO_2_ can also be added in very small quantities (ppm range) to increase arc stability [26]. In addition, the waviness of side surfaces and the mechanical properties of walls can be influenced by the shielding gas [27].

Nitrogen is also used as a shielding gas component in the fusion welding of Ni-based superalloys to affect the weld metal metallurgically. In the case of the high-temperature superalloy Alloy 602 CA (NiCr25FeAlY), which has good high-temperature strength up to 1200 °C due to the precipitation of primary Cr carbides, the addition of nitrogen in the shielding gas leads to a significant reduction in the hot cracking tendency in weld metal [28,29]. Nitrogen exerts a positive effect on carbide morphology in unidirectional and single-crystal Ni-based alloys, as reported by [30,31]. Furthermore, with increasing N content in the shielding gas, an increase in room temperature strength and hardness of the GTAW weld metal of Alloy 263 and Alloy X can be observed [32].

Therefore, the present paper informs about the influence of arc energy and shielding gas during WAAM of wall and block parts with HY282 on cooling times, macro- and microstructures as well as mechanical-technological properties at room temperature and elevated temperature. The tests were performed in the welded and partially precipitation-hardened condition. Different geometries were used to generate various welding conditions (one bead/layer walls or multi-layer deposits with stringer beads) and residual stress conditions. The weld deposits were carried out with different wire diameters of HY282, since not enough material was available in one diameter.

## 2. Experimental Setup

### 2.1. Filler Metal

A batch of the solid wire electrode HY282 in two different diameters (0.040″ and 0.035″) was available for the investigations. Table 1 shows the target chemical composition by the wire manufacturer [33] and the actual chemical composition of the wire batch used.

This was determined using atomic emission spectrometry with Spectrolab S (SPECTRO Analytical Instruments GmbH) on buttons melted from the solid wire electrodes using the Arc Melter MAM-1 (Edmund Bühler GmbH) under 100% argon. The methodology is described in [34]. The contents of C and S were determined on the as-received wire electrodes by combustion analysis using the G4 ICARUS Series 2 (Bruker Corporation).

### 2.2. Setup for WAAM

In this study, the cold metal transfer (CMT) process was selected for gas metal arc welding (GMAW) because of its low arc energy and stable process behavior. The deposits were made in synergic mode using power source CMT Advanced 4000 with RCU 5000 control unit and a three-axis gantry machine with programmable logic controller. All weld beads were produced in flat position (1G) with neutral welding torch orientation and alternating welding direction after each layer. No brushing was done between the layers and the maximal interpass temperature was 100 °C. The electrical welding parameters, the shielding gas flow, and the wire feed speed were additionally recorded with the external measuring system Weld Analyst-S2 (HKS Prozesstechnik GmbH). The cooling time measurements were made with a 1-channel pyrometer (measuring range: 385 to 1600 °C). They were measured directly behind the arc on the seam surface of every second layer of the walls and every second weld bead of the blocks. Due to the absence of a specific temperature interval for Ni-based materials (comparable to the t_8/5_ cooling time for fine-grain structural steels or the t_12/8_ cooling time for CrNi steels), the times for cooling from 1000 to 600 °C were determined. In this range, secondary phases precipitate in many Ni-based alloys. Table 2 gives an overview of the experimental program.

In the first step, the influence of arc energy on the cooling rate, the macro- and microstructure, and the mechanical properties at room temperature (RT) during welding of thin walls was investigated. For this, the wire feed speed (WFS) was varied between 8.0 and 14.0 m/min and the travel speed (TS) between 0.4 and 0.8 m/min. The constant wall height resulted in a different number of beads and wall widths. A selected arc energy was used to produce a multi-layer block with the stringer bead technique.

In the second step, the influence of shielding gases on the abovementioned properties was investigated. Additional to the shielding gas Z-ArHeHC 30/2/0.05, which contains Ar, 30% He, 2%H_2_, and 550 ppm CO_2_, the reducing shielding gas R1-ArHeH30/1 and the 5% N_2_-containing shielding gas Z-ArHeNC 5/5/0.05 were used for the reasons mentioned above.

## 3. Test Methods

The various components were first subjected to a visual and dye penetration test. Metallographic cross sections (Y–Z plane) were then taken. For the automatic preparation, it was necessary to cut the cross sections of walls and blocks in the middle. All samples were processed with standard metallographic techniques with a final polish of 0.5 μm O.P.S (SiO_2_). Subsequently, the central part of the walls and blocks were mechanically machined to allow high-quality X-ray inspection. While the image quality indicator according to [36] on the walls was 16 or 17, only an indicator of 10 was achieved on the blocks due to their higher thickness. For this reason, no radiographic testing was performed on the blocks. On the walls and blocks, chemical analyses, tensile tests, and hardness measurements were performed. Standard tensile tests were performed to BS EN ISO 6892-1 [37] at room temperature. The tensile specimens with form E 5 × 10 × 40 [38] were taken only in welding direction. Vickers hardness testing was measured under a test load of 10 kg and a load dwell time of 8 s in compliance with BS EN ISO 6507-1 [39]. Only on the blocks was the determination of the impact energy on Charpy V specimen at room and low temperatures (−196 °C) carried out. The microstructure of different WAAM parts was investigated using a light optical microscope (Inverse incident light microscope Leica MeF4A, Leica) and scanning electron microscope (XL30 FEG/ESEM, company FEI/Philips).

The post-weld heat treatment (PWHT) was performed in a vacuum chamber furnace from IVA Schmetz GmbH in conjunction with a thermo process management system in a vacuum on final machined specimens. It followed the standard procedure and was performed at 1160 °C/2 h + 1010 °C/2 h + 788 °C/8 h. For accelerated cooling, argon was injected into the furnace and swirled by a large speed-controlled fan. The hot tensile tests at 871 °C were performed with the test machine Zwick Roell Z100 with a 3-zone furnace at atmosphere in compliance with the ISO 6892 [40].

## 4. Results

### 4.1. Process Parameters and Arc Energy

Table 3 shows measured arithmetical values for wire feed rate (WFS), current (I), and voltage (U) as a function of the setting values when deposited walls and block with the standard shielding gas Z-ArHeHC 30/2/0.05. Arc energy (En) is determined in welding as the ratio of arc power (P) to arc travel speed (TS), as in Equation (1).

(1) $$\mathrm{En}=\frac{\bar{P}}{\mathrm{TS}}=\frac{\bar{I}\cdot \bar{U}}{\mathrm{TS}}$$

**Figure 1:**
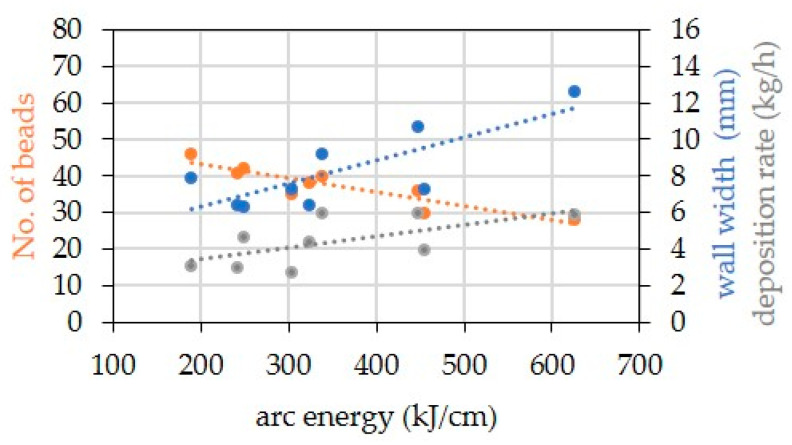
Influence of arc energy on number of layers, walls width, and deposition rate when welded the walls with variable process parameters from Table 2.

The arc energy (En) given in Table 3 was calculated as the arithmetic mean value of the arc energies of the single weld beads. Differences between the nominal and actual wire feed rates occurred for all welds, which affects the deposition rate. This results from the fact that the CMT synergic lines used were not developed for the used wire electrode–shielding gas combinations. In order to achieve a stable process behavior, the welding power source uses an internal control to regulate, for example, the wire feed frequency, which affects the average of the wire feed speed. The deposition rate (DR) is the amount of wire melted per time. At constant wall height, the wall width, the number of layers, and the deposition rate increase with increasing heat energy (Figure 1).

**Figure 2:**
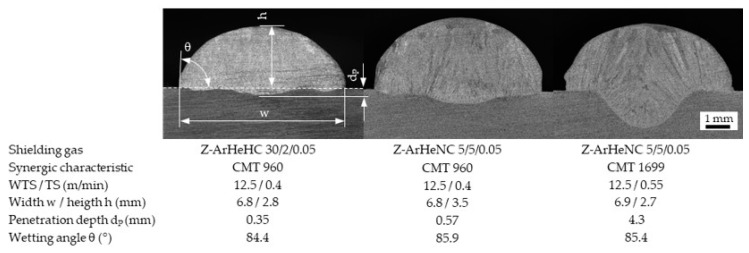
Seam geometries of single beads on the plate (Y–Z plan) welded with different shielding gases and CMT lines.

The shielding gas tests, implemented with the thinner 0.035″ wire diameter of HY 282, required the choice of another CMT reference No. 960 V1.0.0.3.4. However, the use of the nitrogen-containing shielding gas with only 5% helium reduced the arc voltage to such an extent that a more unsteady arc and an unacceptable weld geometry occurred. For this reason, the CMT reference No. 1699 V.4.5.0 was selected for this shielding gas. In order to ensure approximately the same arc energy and weld geometries, the welding speed was increased from 0.4 to 0.55 m/min. As can be seen in Figure 2, the cross sections of single beads show a similar seam geometry. Only the penetration depth increases significantly when using CMT 1699.

Table 4 shows the arithmetic mean values for process parameters when welding with different CMT synergic lines and shielding gases. The higher average wire feed speeds and welding currents result from the CMT 1699 synergic line, which differs significantly in its electrical waveform from the CMT 960 synergic line.

**Figure 3:**
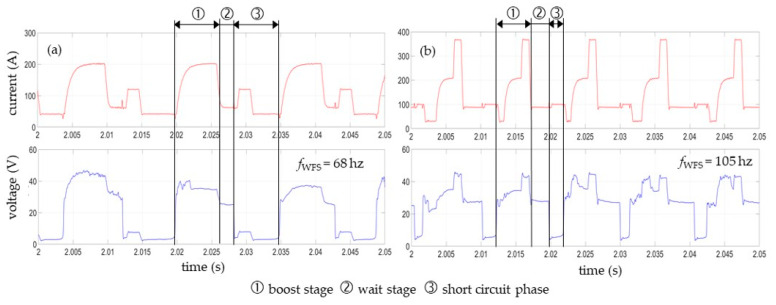
Typical electrical waveform of the CMT cycles for (**a**) CMT 960 and (**b**) CMT 1699.

A CMT cycle consists of a plasma phase (composed of boost and wait stage) and the short-circuit phase. In the boost stage, the filler wire and base material are melted, producing the molten droplet and the weld pool. During the short-circuit phase, the electromagnetic pinch effect causes the molten droplet to constrict at the tip of the wire electrode. For the CMT 960 synergic line, this process is supported by a small current pulse of about 120 A (Figure 3a). In contrast, with the CMT 1699 synergic line, the welding current initially drops briefly in the boost state, then rises and ends with a targeted, almost vertical peak of approx. 380 A (Figure 3b). In addition to that, the short-circuit time is significantly shorter here, and the frequency oscillating wire feeding (*f*_WFS_) thus the number of CMT cycles per second is higher. This causes the higher welding currents and arc voltages.

### 4.2. Cooling Times Depended on Process Parameters

**Figure 4:**
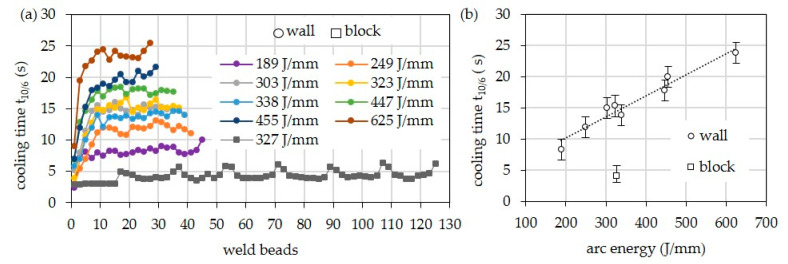
Cooling times of different parts (wall, block) welded with HY282 (Φ 0.04″) and Z-ArHeHC 30/2/0.05 (**a**) depending on arc energy and weld beads and (**b**) arithmetic mean values at 11th weld bead depending on arc energy.

Figure 4 shows the cooling times t_10/6_ for the walls, depending on arc energy and layer. In the first layers, shorter cooling times occur due to the still-possible heat conduction into the substrate. Starting from the ninth layer, the 3D heat conduction changes to a 2D heat conduction, which is why the cooling time remains almost constant. As expected, the cooling times increase with increasing arc energy when welding the walls. At the highest arc energy of 625 J/mm, the cooling time is about 24 s. At the lowest arc energy of 189 J/mm, the cooling time is only 8 s. In the case of the multi-pass block, the average cooling time is only approx. 4.1 s due to the 3D heat conduction. It is thus significantly shorter than that of the bar with comparable arc energy.

**Figure 5:**
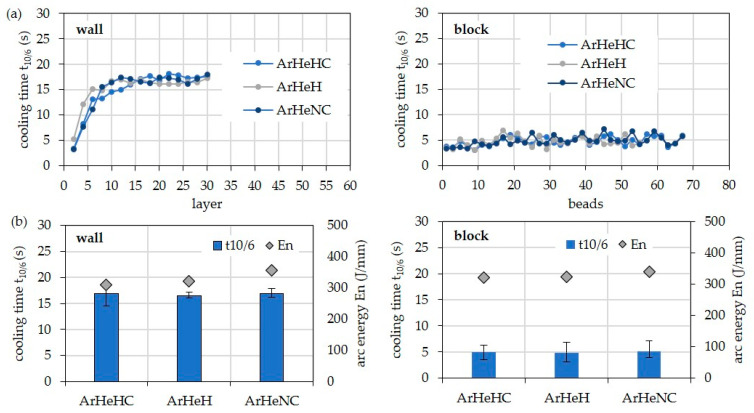
Cooling times of different parts (wall, block) welded with HY282 (Φ 0.035″) depending on shielding gas. (**a**) Single values over the layers. (**b**) Arithmetic mean values from 10th layer.

Independent of the structure, the shielding gas shows only a minor influence on the cooling time since the values for arc energy do not differ significantly (Figure 5).

### 4.3. Non-Destructive Testing and Chemical Analysis

**Figure 6:**
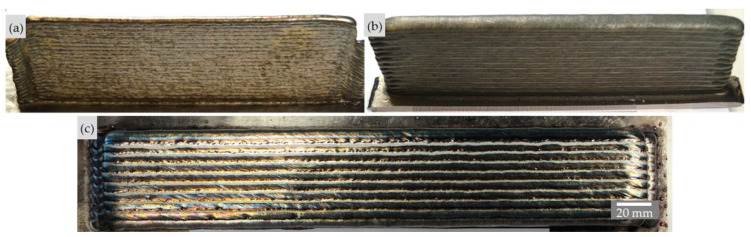
Side views of brushed walls. (**a**) WFS: 8.0 m/min, TS: 0.8 m/min; (**b**) WFS: 11.0 m/min, TS: 0.6 m/min; (**c**) top view of the unbrushed block with WFS: 14.0 m/min, TS: 0.4 m/min (Z-ArHeHC).

**Figure 7:**
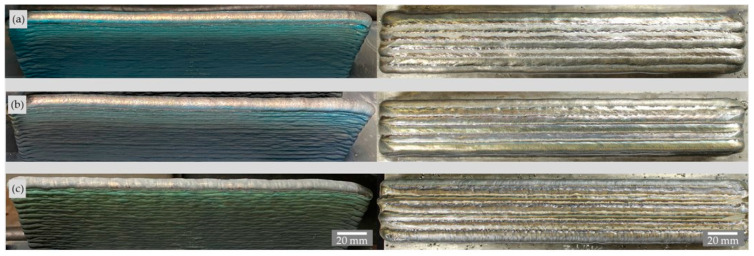
Side view of the unbrushed walls (**left**) and top views of the brushed blocks (**right**). (**a**) Z-ArHeHC; (**b**) R1-ArHeH; (**c**) Z-ArHeNC (welding parameters in Table 4).

Visual and penetrant inspections of all printed walls and blocks did not reveal any external defects such as cracks, lack of fusion, or gas pores in the weld metal. Figure 6 shows two walls with low and high arc energy and the 330 mm long block. The wall produced with the higher arc energy has a dull, slightly more oxidized surface than the wall produced with the lower arc energy because the melt pool dimensions and cooling times are higher here. Figure 7 shows the walls and the blocks produced with the different shielding gases. The walls, which are still unbrushed, show the metal vapor deposits with different colors resulting from the welding process. The block produced with the N shielding gas shows inhomogeneous fusion lines between the single stringer beads.

The X-ray examinations of all walls showed no or only very small gas porosity of less than 0.001%. No radiographic tests were performed on the blocks due to the low image quality number as they did not provide sufficient information.

**Figure 8:**
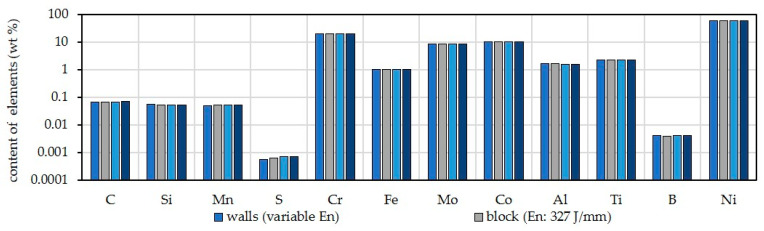
Arithmetic mean values of chemical compositions of HY282 walls and blocks depending on arc energy and shielding gas (scale of y-axis: log to base 2).

Since a shielding gas with low active and reducing shielding gas components was used and no brushing between the individual layers took place, the chemical compositions of weld metals were determined. For the evaluation, arithmetic mean values were used for the individual alloying elements of the various wall and block structures, which were produced with varying arc energy and shielding gases. As shown Figure 8, no pickup or burning loss of alloying elements has occurred.

Carrier gas melt extraction (G8 Galileo, Bruker Corporation) was also used to measure the contents of oxygen and nitrogen of walls and blocks. The difference in gas content between weld metal and the wire electrode was determined using Equation (2).

Δ_X_ = X_weld metal_ − X_wire_ (2)

X_wm_: Content of O or N in weld metal (wm)

X_wire_: Content of O or N in wire electrode (wire)

**Figure 9:**
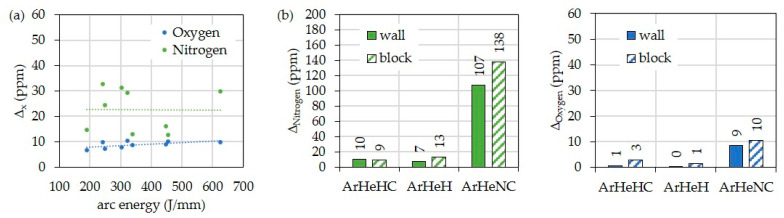
Increase of oxygen and nitrogen in the walls and blocks compared to the wire electrode HY282 depending on (**a**) arc energy (Φ 0.040″) and (**b**) shielding gas (Φ 0.035″).

In general, a slight increase in oxygen and nitrogen can be seen for all weld metal types. However, there is no significant influence of the arc energy (Figure 9a). On the other hand, the use of the shielding gas Z-ArHeNC 5/5/0.05 causes a significant increase in the nitrogen content of the weld metal (Figure 9b). The oxygen values are also slightly higher (Figure 9c).

### 4.4. Macrostructure

**Figure 10:**
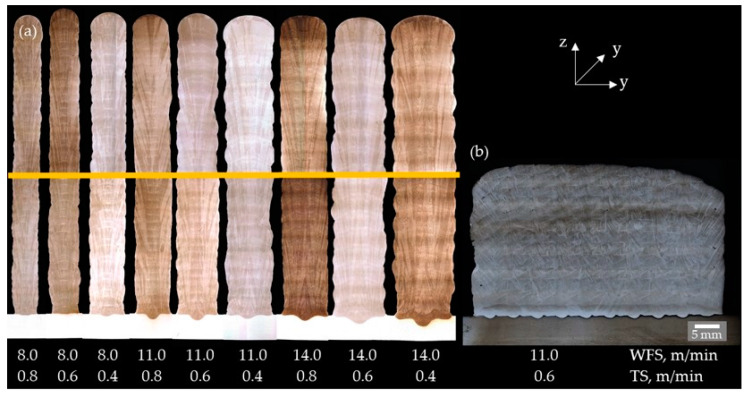
Macro cross sections (Y–Z plane) of walls (**a**) and block (**b**) welded with HY282 (Φ 0.04″) depending on arc energy (etched with Beraha III).

The cross sections of the walls in Figure 10 showed no unacceptable internal seam irregularities such as lack of fusion, pores, or hot cracks. In the multi-pass block, however, three micro hot cracks occurred with a total crack length of 810 µm and an *MSI* of 0.47 µm/mm^2^.

The *MSI* (micro fissure sensitivity index) is the quotient of total micro fissure length and the weld metal area. Comparable block welds with Ni alloy Inconel 718 showed a significantly higher hot cracking occurrence with an *MSI* between 2.32 and 33.3 [25].

**Figure 11:**
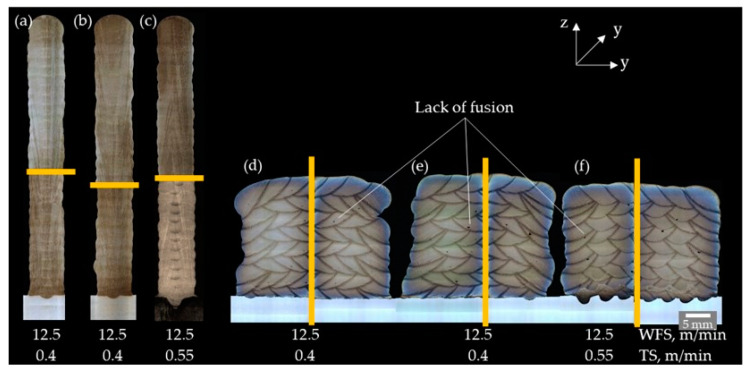
Macro sections (Y–Z plane) of walls (**left**) and blocks (**right**) with HY282 (Φ 0.035″) depending on shielding gases. (**a**,**d**) ArHeHC; (**b**,**e**) ArHeH; (**c**,**f**) ArHeNC (wall: etched with Beraha III, block: electrolytic etched with 10% CrO_3_ and 90% water).

The macro sections of the walls and blocks deposited with different shielding gases showed similar behavior (Figure 11) The walls exhibited no seam defects, but in the blocks, unacceptable lack of fusion defects and a few hot cracks occurred. The lack of fusion was caused by the bulging stringer weld beads, the inhomogeneous fusion lines, and the excessive lateral welding torch feed. The shielding gas influenced the hot cracking occurrence in the blocks as follows:

- Z-ArHeHC: six hot cracks, 1080 µm total crack length, 2.50 µm/mm^2^ MSI
- R1-ArHeH: two hot cracks, 390 µm total crack length, 1.05 µm/mm^2^ MSI
- Z-ArHeNC: no hot cracks.

**Figure 12:**
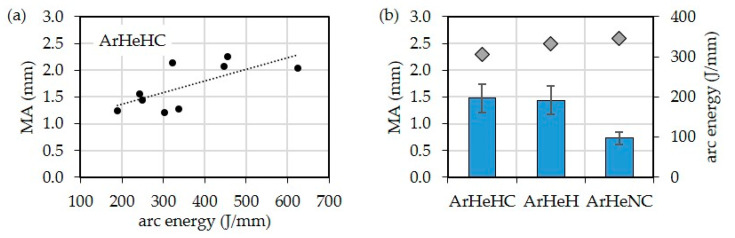
Machining allowance *MA* of walls depended on (**a**) arc energy and (**b**) shielding gas.

In order to determine the influence of the welding parameters on the surface waviness of the walls, the machining allowance *MA* on the cross sections (Y–Z plane) of the walls was determined by image analysis. This allowance would be required to mill a flat structure from the wall. It is therefore the dimensional difference between the finished dimension and the initial dimension of the wall width, e.g., for subsequent milling. It can be seen that the surface waviness increases with increasing wire feed speeds. Figure 12 shows that higher arc energy has an unfavorable effect on the surface waviness. With respect to the shielding gases influence, the walls manufactured with ArHeNC and CMT1699 synergic line have the lowest waviness.

**Figure 13:**
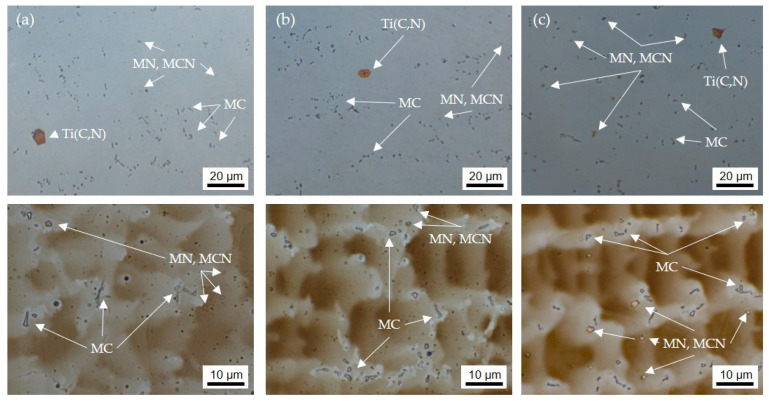
Micro sections (Y–Z plane) of walls with HY282 (Φ 0.035″). (**a**) ArHeHC; (**b**) ArHeH; (**c**) ArHeNC (**top**: polished, **bottom**: etched with Beraha III).

Figure 13 exemplifies the microstructures of the last deposited layer of the walls produced with different shielding gases. The additive weld metal exhibits the typical dendritic solidification microstructure with interdendritic precipitated MC described in the literature [8,9] for welded HY282. Additionally, isolated large orange particles are visible which are classified as Ti(C,N) on the basis of their color and geometry. There are many small angular yellow particles which are probably MN-type nitrides or MCN-type carbonitrides, where M can stand for Ti, Al, and/or B due to the given chemical composition of HY282. These elements are known to have a high affinity for nitrogen and optionally carbon. As expected, the amount of these particles is greater in the weld metal deposited with ArHeNC due to the higher N content.

### 4.5. Mechanical Properties at Room Temperature

Table 5 and Table 6 show the results of quantitative tensile tests at room temperature (RT) for the wall and block parts depending on the arc energy and the shielding gas used.

**Figure 14:**
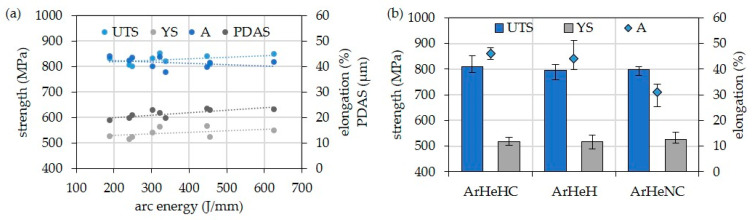
Results of tensile tests at RT of walls of HY282 depended on (**a**) arc energy and (**b**) shielding gas.

As can be seen in Figure 14, the strengths (UTS, YS) and the primary dendrite arm spacing (PDAS) increase slightly with increasing arc energy, i.e., with slower cooling. On the other hand, the elongation at break is slightly reduced. The block shows higher tensile and 0.2% yield strength at the same setting values (WFS: 11.0 m/min, TS: 0.6 m/min) despite faster cooling. Only this structure achieves the typical tensile data specified by the manufacturer for all weld metal (UTS: 860 MPa, 0.2% YS: 586 MPa, A: 40.0%) [41]. However, the scatter of the single values (see Table 5) should also be considered when making these observations. With regard to the shielding gases, it should be noted that the use of ArHeHC and ArHeH lead to comparable mechanical properties. The use of nitrogen in the shielding gas causes increased hardness, lower Charpy V values, and a significant reduction in elongation (see Table 6). Here is an influence of the precipitated MN-type nitrides or MCN-type carbonitrides suspected.

### 4.6. Precipitation Hardening

**Figure 15:**
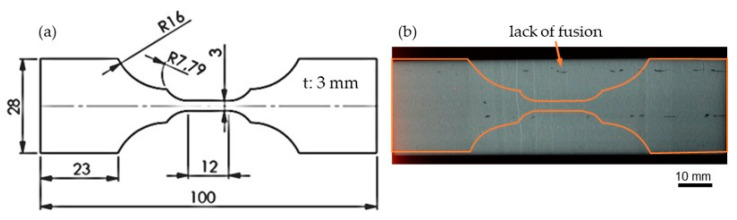
(**a**) Geometry of hot tensile specimens. (**b**) X-ray images of a flat specimen of block with Z-ArHeNC-5/5/0.05 digitized with a reflex camera.

Post-weld heat treatment (PWHT) was performed on specimens from the blocks welded with variable shielding gases. Since the cross sections of these blocks show lack of fusion defects, 3 mm thick flat specimens were first eroded from the block parts, which were then X-rayed. Figure 15a shows hot tensile specimen dimensions, and Figure 15b shows an exemplary radiographed flat specimen. The tensile specimens were then arranged in a way that the test resides in a defect-free part of the specimen.

**Figure 16:**
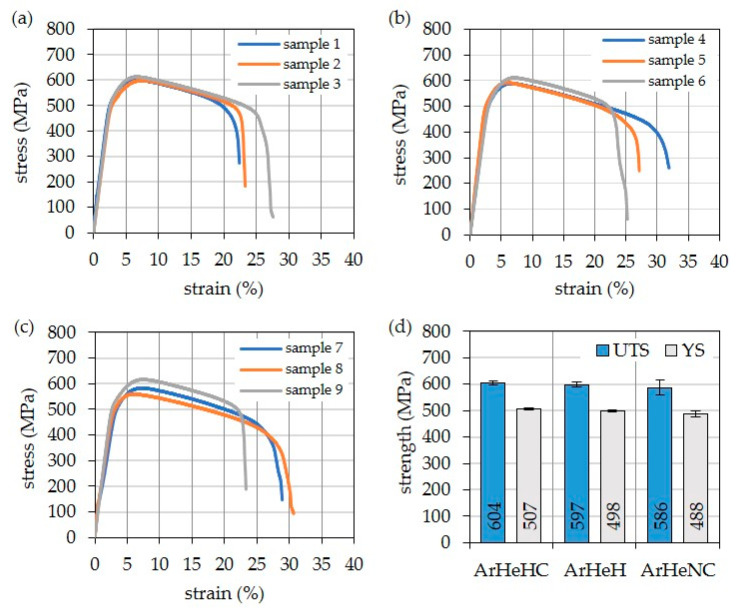
Results of hot tensile tests at 871 °C. (**a**) ArHeHC; (**b**) ArHeH; (**c**) ArHeNC; (**d**) comparison of the arithmetical mean values.

The results of tensile testing at 871 °C of post-welding heat treated specimens are presented in Figure 16. Three specimens were tested in each case. The weld metal produced with Z-ArHeHC shows the highest strength. In contrast, the strengths of the weld metal produced with the nitrogen-containing shielding gas are slightly lower. In the latter case, however, the values are more scattered. All values reach the strength specified by the material manufacturer [41] (UTS: 565 MPa and 0.2% YS: 490 MPA) for a 12.7 mm plate welded with GMAW and a wire with a diameter of 0.045″ (fracture location: weld).

**Figure 17:**
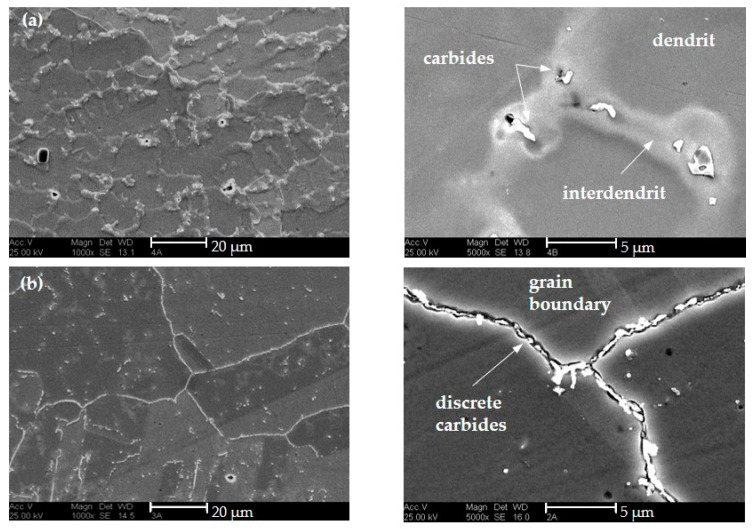
SEM micrographs of specimens, welded with shielding gas Z-ArHeHC. (**a**) as-built; (**b**) precipitation-hardened (etching solution: 5 g C_2_H_2_O_4_ dissolved in 95 mL HCl).

A metallographic evaluation was only possible for specimens produced with the standard gas since no material was available from the other samples. Figure 17 shows the microstructure in the as-built and precipitation hardened stage. While the carbides are primarily interdendritic in the post-deposited state, PWHT also causes precipitation of many discrete carbides at the grain boundaries and within the austenite grains. The appearance of the carbides is similar to the (Ti,Mo)C (as-built) and (Cr, Mo)_23_C_6_ (precipitation-hardened) type carbides described in [8,9,19,42].

**Figure 18:**
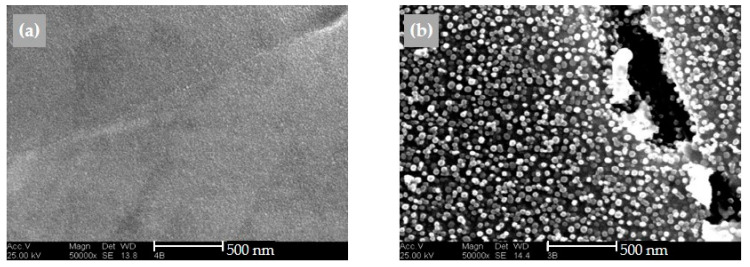
SEM micrographs of specimens, welded with shielding gas Z-ArHeHC. (**a**) as-built; (**b**) precipitation hardened (etching solution: 15 g CrO_3_ + 150 mL H_3_PO_4_ + 100 mL H_2_SO_4_).

In addition to the carbides, precipitation of the γ-phase occurs, as expected (Figure 18). A large number of these γ′-particles are in the size range between 60 nm to 80 nm. The precipitation-hardened weld metal has a higher hardness (346 HV10) than the weld metal (278 HV10).

## 5. Conclusions

1. The results show the promising potential of nickel-based superalloy HAYNES^®^ 282^®^ for the WAAM of voluminous parts of medium to low complexity. The problem of the occurrence of hot cracks during arc welding, which is typical for fully austenitic Ni-alloys, only manifests itself to a limited extent in this alloy. It is only observed within the multi-track blocks due to the stronger thermo-mechanical reactions compared to the walls. However, compared to Inconel 718, it is significantly lower.

2. The CMT process showed a very good suitability for additive welding of walls (one bead/layer) of HY282. However, with the multi-track blocks there is a risk of the occurrence of lack of fusion defects. Here, the use of the CMT impulse process is recommended because it leads to flatter weld beads with a lower wetting angle.

3. The mechanical properties at RT of the produced WAAM weld metal of the walls and blocks are slightly influenced by both the arc energy and the shielding gas. A higher arc energy results in slightly greater strength values and lower elongation. Within the shielding gases the N-containing shielding gas (Z-ArHeNC 5/5/0.05) results in higher hardness and in lower notched bar impact energy and elongation.

4. The results of tensile testing at 871 °C of post-welding heat treated specimens give good strength properties independent of the shielding gas. They all achieve the strength specified by the material manufacturer [41]. However, the weld metal produced with Z-ArHeHC shows the highest strength. In contrast, the strengths of N-containing weld metal are slightly lower.

5. The carbides observed in the weld metals correspond in appearance and location to those described in [8,9,19,42]—(Ti,Mo)C in the as-built condition and (Cr, Mo)_23_C_6_ in the precipitation hardening condition. In addition, large block-like nitrides and small nitride precipitates are visible. The latter occur significantly more frequently in the weld metal of Z- Z-ArHeNC 5/5/0.05.

## Figures and Tables

**Table 1:** Chemical composition of solid wire electrodes HY282 (wt%).

Analysis	Φ, ″	C	Si	Mn	S	Cr	Fe	Mo	Co	Al	Ti	B	Ni
Target	-	0.04–0.08	≤0.15	≤0.3	≤0.015	18.5–20.5	≤1.5	8–9	9–11	1.38–1.65	1.9–2.3	0.003–0.010	bal.
Actual	0.040	0.065	0.05	0.05	0.0006	19.23	0.99	8.15	10.05	1.59	2.20	0.0039	57.45
	0.035	0.065	0.05	0.05	0.0006	19.23	0.99	8.16	10.10	1.59	2.26	0.0040	57.32

**Table 2:** Experimental program for generation of different WAAM parts with HY282 with variable arc energy and shielding gases.

Step	Criterion	Φ (″)	CMT	Shielding Gas	Setting Values (m/min)		Structure	Dimensions (mm)		
			Reference No.	(acc. to [35])	WFS	TS		L	H	W
1.	Arc energy	0.040	1254 V2.3.8.4	Z-ArHeHC 30/2/0.05	8.0; 11.0; 14.0	0.4; 0.6; 0.8	wall	225	65	variable
	Structure	0.040	1254 V2.3.8.4	Z-ArHeHC 30/2/0.05	11.0	0.6	block	330	32	55
2.	Shielding gas	0.035	960 V1.0.0.3.4	Z-ArHeHC 30/2/0.05 R1-ArHeH30/1	12.5	0.4	wall	180	60	variable
							block	220	27	30
			1699 V4.5.0	Z-ArHeNC 5/5/0.05	12.5	0.55	wall	180	60	variable
							block	220	28	35

**Table 3:** Process parameters of different WAAM parts with HY282 (Φ 0.04″) and shielding gas Z-ArHeHC 30/2/0.05 (flow rate: 18 L/min; stick-out: 12 mm).

Process Parameter			Wall									Block
Setting	WFS	m/min	8.0	8.0	8.0	11.0	11.0	11.0	14.0	14.0	14.0	11.0
	TS	m/min	0.4	0.6	0.8	0.4	0.6	0.8	0.4	0.6	0.8	0.6
Measured	$\bar{\mathrm{WFS}}$	m/min	6.5 ± 0.2	7.1 ± 0.5	7.4 ± 0.3	9.3 ± 0.3	10.5 ± 0.4	11.0 ± 0.4	13.9 ± 0.4	14.2 ± 0.2	14.1 ± 0.2	10.7 ± 0.5
	$\bar{I}$	A	117 ± 3.4	124 ± 4.2	127 ± 3.1	145 ± 3.5	153 ± 6.5	152 ± 8.6	181 ± 5.6	188 ± 7.4	188 ± 7.8	159 ± 8.2
	$\bar{U}$	V	17.3 ± 0.9	19.6 ± 2.0	19.8 ± 2.2	20.9 ± 0.8	21.2 ± 1.6	22.0 ± 2.5	23.1 ± 0.7	23.8 ± 1.0	24.0 ± 1.2	20.6 ± 2.0
	$\bar{\mathrm{En}}$	J/mm	303 ± 12	242 ± 28	189 ± 21	455 ± 17	323 ± 20	249 ± 24	625 ± 25	447 ± 16	338 ± 13	327 ± 27

**Table 4:** Process parameters of different WAAM structures with HY282 (Φ 0.035″) and variable CMT synergic lines and shielding gases (flow rate: 18 L/min; stick-out: 12 mm).

Shielding Gas					Synergic Line	Structure	Setting Values		Measured Values				
Ar	He	H_2_	N_2_	CO_2_	Short Name		WFS	TS	$\bar{WFS}$	$\bar{I}$	$\bar{U}$	$\bar{En}$	*DR*
Bal.	30	2	-	0.055	CMT 960 ArHeHC	wall	12.5	0.40	10.0 ± 0.3	114 ± 1.8	18.0 ± 0.4	309 ± 11	3.1
						block	12.5	0.40	11.0 ± 1.0	118 ± 1.7	18.2 ± 1.4	321 ± 27	3.4
Bal.	30	1	-	-	CMT 960 ArHeH	wall	12.5	0.40	11.7 ± 0.7	115 ± 1.1	19.3 ± 0.7	333 ± 12	3.6
						block	12.5	0.40	11.1 ± 1.1	118 ± 1.7	18.6 ± 1.3	322 ± 23	3.4
Bal.	5	-	5	0.055	CMT 1699 ArHeNC	wall	12.5	0.55	14.1 ± 0.3	151 ± 3.2	21.5 ± 04	356 ± 8.9	4.4
						block	12.5	0.55	13.5 ± 0.9	152 ± 2.6	20.4 ± 1.1	339 ± 14	4.1

**Table 5:** Mechanical properties of wall and block parts of HY282 (Φ 0.04″) depending on arc energy.

Symbol	Unit	Wall									Block
WFS	m/min	8.0	8.0	8.0	11.0	11.0	11.0	14.0	14.0	14.0	11.0
TS	m/min	0.4	0.6	0.8	0.4	0.6	0.8	0.4	0.6	0.8	0.6
UTS	MPa	832 ± 4	807 ± 26	833 ± 5	810 ± 3	851 ± 49	802 ± 12	849 ± 26	840 ± 12	821 ± 9	879 ± 10
0.2% YS	MPa	540 ± 3	514 ± 15	528 ± 2	523 ± 22	563 ± 24	525 ± 8	549 ± 8	567 ± 14	558 ± 5	594 ± 29
A	%	40.1 ± 2.5	42.3 ± 1.1	44.2 ± 0.5	41.6 ± 1.6	43.8 ± 0.7	43.6 ± 1.1	41.8 ± 1.2	39.7 ± 2.4	37.7 ± 5.1	43.8 ± 1.7
Hardness	HV10	267 ± 9	264 ± 9	267 ± 9	261 ± 7	276 ± 11	268 ± 8	263 ± 11	267 ± 9	277 ± 13	271 ± 13

**Table 6:** Mechanical properties of wall and block parts of HY282 (Φ 0.035″) depending on shielding gas.

Structure	Parameter	Unit	ArHeHC	ArHeH	ArHeNC
wall	UTS	MPa	810 ± 30	796 ± 25	799 ± 16
	0.2% YS	MPa	516 ± 13	517 ± 22	527 ± 19
	A	%	46.0 ± 1.8	44.0 ± 5.0	31.1 ± 4.1
	Hardness	HV10	276 ± 16	265 ± 8	293 ± 13
block	KV_2_ at RT	J	144 ± 4	142 ± 3	133 ± 9
	KV_2_ at −196 °C	J	133 ± 7	129 ± 1	126 ± 0
	Hardness	HV10	278 ± 18	270 ± 14	296 ± 18

## Data Availability

The data presented in this study are available in the article.
